# Torque-producing capacity is affected by moment arm in the human knee extensors

**DOI:** 10.1186/s13104-020-05182-3

**Published:** 2020-07-20

**Authors:** Miyuki Hori, Tadashi Suga, Masafumi Terada, Yuto Miyake, Akinori Nagano, Tadao Isaka

**Affiliations:** Faculty of Sport and Health Science, Ritsumeikan University, 1-1-1 Nojihigashi, Kusatsu, Shiga 525-8577 Japan

**Keywords:** Isometric torque, Muscle volume, Knee extension, Quadriceps femoris, Magnetic resonance imaging

## Abstract

**Objective:**

The torque-producing capacity can be assessed as maximal isometric torque per muscle size. Nevertheless, the factors contributing to this capacity remain poorly understood. In general, the magnitude of joint torque production is determined not only by muscle size but also by joint moment arm (MA). Based on this background, we hypothesized that longer MA would be related to higher torque-producing capacity despite a given muscle size. To test this hypothesis, we examined the relationship between MA and toque-producing capacity in the knee extensors. The quadriceps femoris muscle volume (MV) and knee extensor MA in 30 healthy young men were measured using magnetic resonance imaging. The knee extensor isometric torque was measured using a dynamometer. The knee extensor torque-producing capacity was calculated as the knee extensor isometric torque per quadriceps femoris MV.

**Results:**

The quadriceps femoris MV and knee extensor MA correlated significantly with the knee extensor isometric torque (*r* = 0.785 and 0.790, respectively, both *P*s < 0.001). Furthermore, the knee extensor MA correlated significantly with the knee extensor torque-producing capacity (*r* = 0.635, *P* < 0.001). These findings suggest that longer MA is an important factor for achieving higher torque-producing capacity in the human knee extensors.

## Introduction

Maximal isometric torque per agonist muscle size is considered as the torque-producing capacity [1], which is also called specific strength or muscle quality [2–5]. This capacity is often associated with several factors, including physiological and neurological factors [3, 4, 6–8]. Previous studies demonstrated that toque-producing capacity was higher in younger subjects than in older subjects [3, 7, 8]. By contrast, other studies reported that torque-producing capacity was comparable between younger and older subjects [2, 9–11]. Additionally, Fukunaga et al. [11] reported that toque-producing capacity did not differ between trained and untrained subjects. They indicated that when several morphological factors such as the joint moment arm (MA), fascicle length, and pennation angle were identical, torque-producing capacity remained unchanged.

The magnitude of joint torque is morphologically defined as the product of agonist muscle size and joint MA dimension. Previous studies demonstrated that a positive correlation between MA and isometric torque in several joints [12–14]. Blazevich et al. [13] determined that although knee extensor MA correlated with knee extensor isometric torque, the knee extensor MA ranged from 33.2 to 47.2 mm. Baxter and Piazza [12] reported that although plantar flexor MA correlated with plantar flexor isometric torque, a difference between the subjects having the shortest and longest plantar flexor MAs was about 20 mm. These findings suggest that the joint MA dimension is different among subjects in a single population. Thus, despite a given muscle size, subjects having a greater MA can be more enhanced torque-producing capacity than subjects having a smaller MA. Based on these backgrounds, we hypothesized that longer MA would be related to higher torque-generating capacity among a single population. To test this hypothesis, the present study examined the relationship between MA and torque-generating capacity of the knee extensors in healthy adults.

## Main text

### Methods

#### Subjects

Thirty healthy young men (age: 21.9 ± 1.8 years, body height: 171.6 ± 5.4 cm, body weight: 66.0 ± 7.9 kg) participated in this study. The subjects were recreationally active but were not involved in any specific physical training program within the previous 3 years. Nevertheless, many of them had participated in recreational sports and/or physical training for 2–3 h per week. Informed written consent was obtained from all subjects. All subjects had no history of orthopedic injuries or previous surgery of the knee extensors, and were free of any known neurologic, cardiovascular, or pulmonary disorders. This study was approved by the Ethics Committee of Ritsumeikan University.

#### Quadriceps femoris muscle volume (MV) and knee extensor MA

**Fig. 1:**
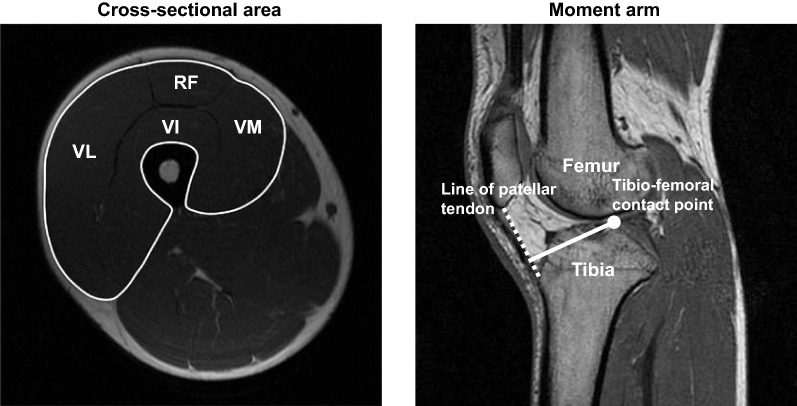
Representative magnetic resonance imaging scans of mid-thigh quadriceps femoris cross-sectional area and knee extensor moment arm. The quadriceps femoris cross-sectional area included the rectus femoris (RF), vastus intermedius (VI), vastus lateralis (VL), and vastus medialis (VM). The quadriceps femoris muscle volume was calculated by multiplying the sum of the cross-sectional areas along their length at intervals of 10 mm. The knee extensor moment arm was calculated as the distance between the tibio-femoral contact point and the mid-line of the patellar tendon

Representative images of the mid-tight quadriceps femoris cross-sectional area (CSA) and knee extensor MA on magnetic resonance imaging (MRI) are shown in Fig. 1. The methods of MRI measurements of both axial and sagittal images for circulating the quadriceps femoris size and knee extensor MA of the right leg have been previously described [15, 16]. The quadriceps femoris MV was calculated by multiplying the sum of the CSAs along their length at intervals of 10 mm. The knee extensor MA was calculated as the distance between the tibio-femoral contact point and the mid-line of the patellar tendon [13, 15, 16]. The analyses for measuring the quadriceps femoris MV and knee extensor MA were conducted using image analysis software (OsiriX Version 5.6, Switzerland).

#### Knee extensor isometric torque and torque-producing capacity

The knee extensor isometric torque of the right leg was measured using a dynamometer (BIODEX system 3; BIODEX Medical, Shirley, NY, USA). Subjects were secured at the hip by a seat belt to prevent any movement of the hip joint. The hip and knee joint angles were fixed at 100° and 90° (full extension was at 180°), respectively. The ankle joint was attached to a bar connected to the force transducer. A 90° of the knee angle during isometric contraction was selected as commonly adopted in previous studies [8, 13, 14, 17, 18]. The two trials for measuring isometric torque were each performed for 3 s with a 1-m rest period. If the difference between the torque values of the two trials was more than 5% of the highest value, additional trials were performed until this was corrected [18]. The highest value of the two trials, or more than two trials, was used as the knee extensor isometric torque. The knee extensor torque-generating capacity was calculated as the knee extensor isometric torque per quadriceps femoris MV.

#### Statistical analysis

The data are presented as the mean ± SD. Relationships between variables were evaluated using a Pearson’s product moment correlation. To adjust for confounding factors, relationships between morphological parameters (i.e., the quadriceps femoris MV and knee extensor MA) and isometric torque of the knee extensors were examined using partial correlation analyses after controlling for body size. This model was adjusted for both body height and body weight. The level of statistical significance was defined at *P* < 0.05. All statistical analyses were conducted using SPSS software (version 19.0; IBM Corp, Armonk, NY, USA).

### Results

Mean values of measured variable in subjects were 2015.4 ± 288.5 (range, 1453.0 to 2488.2) cm^3^ for the quadriceps femoris MV, 43.8 ± 2.4 (range, 40.0 ± 48.8) mm for the knee extensor MA, 244.8 ± 49.8 (range, 158.6 to 357.5) Nm for the knee extensor isometric torque, and 0.121 ± 0.015 (range, 0.099 to 0.156) Nm/cm^2^ for the knee extensor torque producing capacity.

Body height correlated significantly with the quadriceps femoris MV and knee extensor MA (*r* = 0.420 and 0.456, respectively, both *P*s < 0.05). Body weight also correlated significantly with the quadriceps femoris MV and knee extensor MA (*r* = 0.567 and 0.456, respectively, both *P*s < 0.05). Additionally, there was a significant correlation between the quadriceps femoris MV and knee extensor MA (*r* = 0.577, *P* = 0.001).

**Fig. 2:**
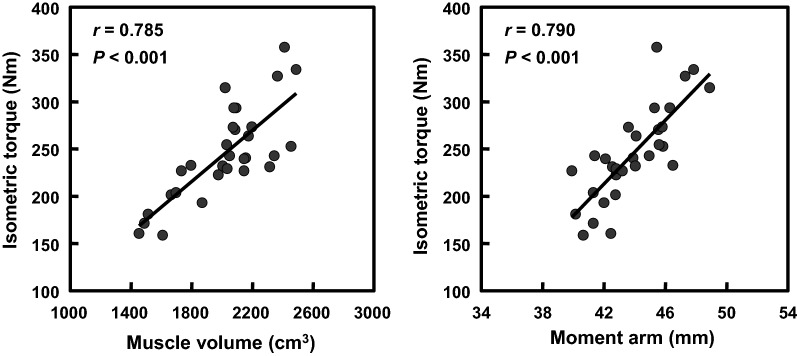
Relationships of the quadriceps femoris muscle volume (right) and knee extensor moment arm (left) with knee extensor isometric toque

Relationships of the quadriceps femoris MV and knee extensor moment arm with knee extensor isometric toque are presented in Fig. 2. The quadriceps femoris MV correlated significantly with knee extensor isometric torque (*r* = 0.785, *P* < 0.001). The knee extensor MA also correlated significantly with knee extensor isometric torque (*r* = 0.790, *P* < 0.001). After adjusting for both body height and body weight, correlations of the quadriceps femoris MV and knee extensor MA with knee extensor isometric torque remained significant (partial *r* = 0.661 and 0.700, respectively, both *P*s < 0.001).

**Fig. 3:**
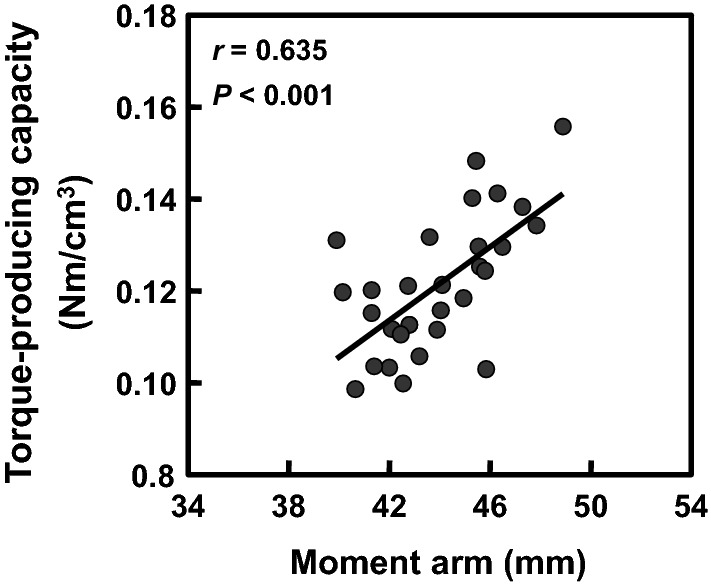
Relationship between knee extensor moment arm and torque-producing capacity. The knee extensor torque-producing capacity was calculated as the knee extensor isometric torque per quadriceps femoris muscle volume

Relationship between the knee extensor moment arm and torque-producing capacity is presented in Fig. 3. The knee extensor MA correlated significantly with knee extensor torque-producing capacity (*r* = 0.635, *P* < 0.001).

### Discussion

This study demonstrated a positive relationship between MA and isometric torque in the knee extensors; thus, this finding corroborates the results of the previous studies [12–14]. Moreover, we determined that after adjusting for body size, such a relationship remained significant, suggesting that the relationship between MA and isometric torque may be independent of the differences in body size among subjects. Furthermore, we found that longer knee extensor MA correlated with higher knee extensor torque-producing capacity. To the best of our knowledge, the present study is the first to examine the relationship between MA and torque-producing capacity and to indicate that torque-producing capacity is affected by MA.

Previous studies determined that torque-producing capacity was higher in younger subjects than in older subjects [3, 7, 8]. Macalusoa et al. [8] reported that torque-producing capacity of the knee extensors was higher in younger subjects than in older subjects. By contrast, a series of studies by Akagi et al. [9, 10] reported that torque-producing capacity of the elbow flexors and extensors were comparable between younger and older subjects. Additionally, Fukunaga et al. [11] reported that the torque-producing capacity of the elbow flexors and extensors did not differ between trained and untrained subjects. Therefore, there is a discrepancy in the previous findings regarding the presence or absence of the difference in torque-producing capacity among several populations. This discrepancy may be attributed to the differences among joints (e.g., elbow vs. knee joints) employed in these previous studies.

In addition to this possibility, Sugisaki et al. [19] examined the difference in the elbow extensor MAs among some elbow extension angles and reported that the mean value of the longest elbow extensor MA among these joint angles in the subjects was 23.9 ± 1.5 mm. This dimension of the elbow extensor MA is nearly 1.8-fold shorter than that of the knee extensor MA (43.8 ± 2.4 mm) measured in the present study. Furthermore, in a study by Sugisaki et al. [19], they reported that the difference between subjects having the shortest and longest elbow extensor MAs was about 4 mm. In the present study, we determined that the difference between subjects having the shortest and longest knee extensor MAs was about 9 mm. Blazevich et al. [13] reported that the difference between subjects having the shortest and longest knee extensor MAs was about 14 mm. In association with the difference in the knee extensor MA among subjects, we found that the difference between subjects having the lowest and highest knee extensor torque-producing capacities was nearly 1.6-fold. Considering these findings, the differences in absolute MA dimension and/or its related joint structure between the elbow and knee joints may be associated with the degree of the difference in torque-producing capacity among several populations. Therefore, we suggest that individuality (i.e., dimension and structure) of the joint among subjects may affect the magnitude of torque-producing capacity of the knee extensors.

The MV is more associated with joint torque than other muscle size parameters such as muscle thickness and anatomical CSA [11]. Additionally, the MV includes components of pennation angle and fascicle length [9, 10]. Nevertheless, the pennation angle and fascicle length may be useful in clearly understanding the present findings; however, the present study did not measure these morphological parameters. Previous studies determined that greater pennation angles of individual muscles among the quadriceps femoris correlated with higher knee extensor isometric torque in healthy adults [14, 17]. Furthermore, previous studies reported a potential relationship between longer fascicle length and higher joint torque [20, 21]. However, whether the magnitudes of pennation angle and fascicle length would be related to torque-producing capacity remains unclear.

Trezise et al. [14] reported that the knee extensor isometric torque correlated with electromyographic activity of individuals muscles among the quadriceps femoris during isometric contraction. Furthermore, previous studies determined that torque-producing capacity may be affected by neuromuscular factors [3, 4, 6–8]. In addition, previous studies reported a potential relationship between torque-producing capacity and muscle fiber composition [22, 23]. To clarify the impact of MA on torque-producing capacity, further studies are needed to examine the individual and interaction effects of morphological, neuromuscular, and physiological factors on torque-producing capacity.

In conclusion, this study demonstrated a positive relationship between MA and torque-producing capacity of the knee extensors in young adults. The present study it the first to determine that longer MA is an important morphological factor for achieving higher torque-producing capacity. In recent studies, we determined that longer knee extensor MA was related to higher sprint performance in sprinters [15, 16], whereas no such a relationship was found with the quadriceps femoris size. Based on the present findings, we hypothesize that sprinters having a longer knee extensor MA can achieve higher sprint performance than sprinters having a shorter knee extensor MA, potentially by enhancing torque-producing capacity, despite a given quadriceps femoris size. To test our hypothesis, further studies are needed to determine the relationships of the knee extensor MA and torque-producing capacity with sprint performance in sprinters.

## Limitation

This study measured the knee extensor MA with both knees fully extended because of a technical limitation of our MRI measurement, as in our and other previous studies [13, 15, 16]. Thus, the joint angle (i.e., 180°) of the knee extensor MA is different from that during isometric contraction (i.e., 90). Nevertheless, previous studies have indicated that the difference in the knee extensor MA at full knee extension (i.e., 180°) among subjects is remained at a joint angle of 90° [24, 25]. Therefore, the present finding can be considered reasonable.

## Data Availability

Data will be provided the corresponding author upon request.

## Abbreviations

CSA: Cross-sectional area
MA: Moment arm
MRI: Magnetic resonance imaging
MV: Muscle volume
